# The use of mesenchymal stem cells for cartilage repair and regeneration: a systematic review

**DOI:** 10.1186/s13018-017-0534-y

**Published:** 2017-03-09

**Authors:** Andy Goldberg, Katrina Mitchell, Julian Soans, Louise Kim, Razi Zaidi

**Affiliations:** 1 Institute of Orthopaedics and Musculoskeletal Science, Royal National Orthopaedic Hospital (RNOH), Brockley Hill Stanmore, London, HA7 4LP UK; 2 Joint Research and Enterprise Office, St George’s University of London and St George’s University Hospitals NHS Foundation Trust, Hunter Wing, Cranmer Terrace, London, SW17 0RE UK

**Keywords:** Matrix-induced autologous chondrocyte implantation, Autologous chondrocyte implantation, Mesenchymal stem cells

## Abstract

**Background:**

The management of articular cartilage defects presents many clinical challenges due to its avascular, aneural and alymphatic nature. Bone marrow stimulation techniques, such as microfracture, are the most frequently used method in clinical practice however the resulting mixed fibrocartilage tissue which is inferior to native hyaline cartilage. Other methods have shown promise but are far from perfect. There is an unmet need and growing interest in regenerative medicine and tissue engineering to improve the outcome for patients requiring cartilage repair. Many published reviews on cartilage repair only list human clinical trials, underestimating the wealth of basic sciences and animal studies that are precursors to future research. We therefore set out to perform a systematic review of the literature to assess the translation of stem cell therapy to explore what research had been carried out at each of the stages of translation from bench-top (in vitro), animal (pre-clinical) and human studies (clinical) and assemble an evidence-based cascade for the responsible introduction of stem cell therapy for cartilage defects.

**Main body of abstract:**

This review was conducted in accordance to PRISMA guidelines using CINHAL, MEDLINE, EMBASE, Scopus and Web of Knowledge databases from 1st January 1900 to 30th June 2015. In total, there were 2880 studies identified of which 252 studies were included for analysis (100 articles for in vitro studies, 111 studies for animal studies; and 31 studies for human studies). There was a huge variance in cell source in pre-clinical studies both of terms of animal used, location of harvest (fat, marrow, blood or synovium) and allogeneicity. The use of scaffolds, growth factors, number of cell passages and number of cells used was hugely heterogeneous.

**Short conclusions:**

This review offers a comprehensive assessment of the evidence behind the translation of basic science to the clinical practice of cartilage repair. It has revealed a lack of connectivity between the in vitro, pre-clinical and human data and a patchwork quilt of synergistic evidence. Drivers for progress in this space are largely driven by patient demand, surgeon inquisition and a regulatory framework that is learning at the same pace as new developments take place.

## Background

Articular cartilage is a highly specialised tissue acting as a shock absorber, enabling synovial joints to articulate with low frictional forces. Due to its avascular, aneural and alymphatic state, it has a limited repair potential [1]. Surgical options to manage damaged articular cartilage include arthroscopic debridement [2–5], bone marrow stimulation techniques [6–8], chondrocyte implantation [9–13], osteochondral autografts (mosaicplasty) [2, 14, 15], osteochondral allograft [16–18] and, in the presence of osteoarthritis, joint replacement [19].

Bone marrow stimulation techniques, such as microfracture, are the most frequently used method in clinical practice for treating small symptomatic lesions of the articular cartilage [6–8]. However, the resulting tissue has shown to be a mixed fibrocartilage tissue [20–22] with varying amounts of type II collagen [8, 21, 23, 24] and inferior to native hyaline cartilage. Fibrocartilage is vulnerable to shear stresses and prone to breaking down over time [20]. Subchondral osseous overgrowth has also been reported after microfracture [25, 26]. Osteochondral grafts can lead to donor site morbidity and healing seams at the recipient site [27, 28]. Autologous chondrocyte implantation (ACI) [9, 10] and its later evolution, matrix-induced autologous chondrocyte implantation (MACI), offered great promise with 80% of patients showing good or excellent results at 10 years [29] but at best results in hyaline-like repair and has experienced complications such as graft failure, periosteal hypertrophy and delamination [30, 31]. In addition, it has also been reported that cells may lose their phenotype during expansion [32, 33].

There is therefore a growing interest in regenerative medicine, which can broadly be thought of as two main types: cell therapy, where cells are injected directly into the blood or into tissues, and tissue engineering, where cell-scaffold combinations are used to repair or regenerate tissues.

Stem cells are cells that have the ability to divide and develop into many different cell types in the body and can be categorised as pluripotent and multipotent. Pluripotent stem cells are often harvested from embryonic sources and can develop into any type of cell in the body whereas multipotent stem cells are generally taken from adults and can divide and develop into a more limited range of cell types. When stem cells divide, the new cells can either remain stem cells or develop into a new type of cell with a more specific function (Table 1).

**Table 1:** Table describing the three main properties of stem cells

Stem cell properties	
• They are unspecialized (“blank slates” that can become specific types of cells).	
• They can develop into specialized cell types (cells that do specific work in the body).	
• They are capable of surviving over long periods and divide to make additional stem cells.	

Mesenchymal stem cells (MSCs) are a form of multipotent cells that may offer an alternative to cartilage repair techniques not hampered by availability and donor site morbidity.

The introduction of stem cell therapies into clinical practice however is a form of translational research, which as per any “bench-to-bedside” pathway now has enormous governance issues [34, 35] and is highly regulatory across four phases (Table 2) and by the Tissues and Cells Directive (2004/23/EC) https://www.hta.gov.uk/policies/eu-tissue-and-cells-directives.

**Table 2:** Description of the different phases of clinical trials

Clinical trial phases (http://www.nlm.nih.gov/services/ctphases.html)	
Phase I: Safety Studies or First-In-Man. Researchers test a new drug or treatment in a small group of people for the first time to evaluate its safety, determine a safe dosage range, and identify side effects.	
Phase II: Uncontrolled Efficacy Studies. The drug or treatment is given to a larger group of people to see if it is effective and to further evaluate its safety.	
Phase III: Randomised Clinical Trials. The drug or treatment is given to large groups of people to confirm its effectiveness, monitor side effects, compare it to commonly used treatments, and collect information that will allow the drug or treatment to be used safely.	
Phase IV: Post-Market Surveillance. Studies are done after the drug or treatment has been marketed to gather information on the drug’s effect in various populations and any side effects associated with long-term use.	

Many published reviews on cartilage repair only list human clinical trials [13, 36–46], underestimating the wealth of basic sciences and animal studies that are precursors to future research and may be relevant in clinical practice further down the line. In addition, true translation would imply that all of the clinical studies would have supporting pre-clinical data.

We therefore set out to perform a systematic review of the literature to assess the translation of stem cell therapy to explore what research had been carried out at each of the stages of translation from bench-top (in vitro)*,* animal (pre-clinical), and human studies (clinical) and assemble an evidence-based cascade for the responsible introduction of stem cell therapy for cartilage defects. In particular, we wanted to focus on the key burning questions pertaining to cartilage repair such as cell source, dosage (how many cells should be used), requirement for scaffolds and the role for extrinsic growth factors.

## Main text

### Search methodology

This review was conducted in accordance to PRISMA guidelines [47] using CINHAL, MEDLINE, EMBASE, Scopus and Web of Knowledge databases from 1st January 1900 to 30th June 2015.

The keywords used in the selection were “(“mesenchymal stem cells”[All Fields] OR “mesenchymal stem cells”[MeSH Terms] OR “mesenchymal”[All Fields] OR “stem cells”[All Fields] OR “Stem Cells”[MeSH Terms] OR “MSC”[All Fields]) AND (“Articular Cartilage”[MeSH Terms] OR “articular”[All Fields] OR “cartilage”[All Fields] OR “cartilage”[MeSH Terms]) AND (“healing”[All Terms] OR “repair”[All Terms] OR “Regeneration”[MeSH Terms] OR “regeneration”[All Fields] OR “tissue engineering”[MeSH Terms] OR “tissue engineering”[All Fields]) AND (“defect”[All Terms]) AND (“chond*”[All Terms])”.

All review and non-English studies were excluded. For analysis, only original research studies were included. Any duplicates were excluded. Initially, KM and JS independently screened studies’ title and abstract. Those included had the full text reviewed. Any disparities were discussed with the senior author (AJG). The references of eligible studies were also searched and included where relevant.

Unpublished trial databases (e.g. ClinicalTrials.gov) were reviewed as the grey literature using popular search engines, including Google. The keywords used for registered clinical trials in clinical trial databases were “stem cells”, “cartilage” and “orthopaedics”.

Eligible studies were drafted into tables tabulating the key data.

### Results

**Fig. 1:**
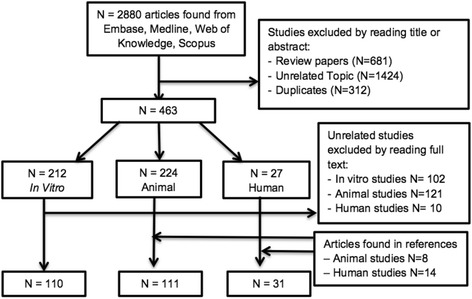
Flow chart of literature search used for the review

The initial search identified 2880 study articles, of which 239 were included for analysis. The PRISMA flow diagram is shown in Fig. 1.

#### In vitro studies

##### MSC source

A list of cell sources used in the in vitro studies is shown in Table 3. The commonest being human MSCs (66%) followed by rabbit MSCs (15%). The majority of the studies used bone marrow-derived MSCs (63%) followed by adipose tissue (33%). Two studies used commercial cell lines [48, 49].

**Table 3:** Cell species and cell sources

Cell species	No. of studies	References	Cell Source	No. of studies^a^	References
Human	73	[48, 50, 52, 53, 168–236]	Bone marrow	62	[48, 50–53, 164, 168, 170–173, 177–180, 182–185, 187, 188, 192, 195–197, 203, 206–210, 212, 216, 217, 219, 221, 223, 227, 230, 232–235, 237–255]
Rabbit	17	[240–242, 246, 249, 252, 255–265]	Adipose	36	[66, 169, 175, 176, 181, 186, 189, 193, 194, 199, 201, 202, 211, 214, 216, 218–220, 224, 228, 229, 231, 235, 242, 256, 257, 260–269]
Bovine	5	[51, 164, 243, 245, 270]	Synovium	9	[174, 191, 200, 213, 222, 226, 258, 259, 270]
Rat/mouse	5	[239, 250, 266, 269, 271]	Umbilical cord blood	3	[205, 236, 190]
Porcine	3	[247, 248, 268]	Commercial cell line	2	[215, 271]
Equine	3	[238, 253, 254]	Placental	2	[198, 225]
Goat	1	[244]	Embryonic	1	[216]
Ovine	2	[237, 251]	Not stated	0	
Not stated	1	[267]			

^a^Some studies used cells from more than one cell source

##### Scaffold

Within the in vitro studies, 26 different types of natural scaffold and 9 types of synthetic scaffolds were identified with a further 18 different types of hybrids, the most popular being a fibrin-polyurethane scaffold (Table 4).

**Table 4:** Types of scaffolds

Number of studies using types of scaffold				
Natural	Synthetic	Hybrid	Growth factor combined	None used
47	14	22	6	29
Scaffold		No. of studies	References	
Types of scaffolds used				
Natural scaffolds				
Type I collagen hydrogel		6	[185, 190, 211, 226, 241, 251]	
Agarose hydrogel		4	[53, 247, 248, 268]	
Alginate bead		3	[223, 231, 271]	
Fibrin hydrogel		3	[208, 211, 263]	
Silk fibroin		3	[198, 216, 256]	
Chitosan microspheres		2	[260, 262]	
Hyaluronic acid		2	[195, 237]	
Cartilage-derived matrix		2	[193, 238]	
K-carrageenan		2	[169, 199]	
Chitosan		2	[168, 216]	
Hyaluronic acid hydrogel		2	[164, 245]	
Gelatin-based scaffold		2	[176, 233]	
Devitalised cartilage ECM		1	[220]	
Bead in bead alginate polysaccharide capsules		1	[221]	
Atelocollagen gel		1	[225]	
Fibrin disk		1	[254]	
Methacrylated hyaluronic acid		1	[164]	
Gelatin microspheres		1	[260]	
Decellularised cell matrix		1	[191]	
Collagen type I microspheres		1	[52]	
Alginate microbeads		1	[266]	
Alginate disks		1	[270]	
Platelet rich plasma		1	[242]	
Free oligosaccharide chondroitin sulphate C		1	[205]	
Collagen type I sponge		1	[237]	
3D printed chitosan		1	[181]	
Synthetic scaffolds				
Polycaprolactone		3	[197, 207, 209]	
PLGA		3	[194, 204, 257]	
Polylactic acid		2	[230, 232]	
PVA		1	[244]	
PGA		1	[178]	
Poly-DL-lactide-co-glycolide		1	[194]	
Polylactide-co-caprolactone		1	[214]	
GFOGER modified PEG hydrogel		1	[183]	
OPF hydrogel		1	[240]	
Hybrid scaffolds				
Fibrin–polurethane hydrogel		4	[50, 188, 192, 267]	
Esterified hyaluronan and gelatin polymer		2	[212, 255]	
TruFit CB (PLGA, calcium sulphate and polycolide)		1	[187]	
PCL–HA bilayer		1	[243]	
PEGDG–crosslinked hyaluronic acid		1	[202]	
Polylactic acid–alginate		1	[232]	
Sodium alginate–hyaluronic acid		1	[189]	
Chitosan–collagen type I		1	[258]	
Polyvinylalcohol–polycaprolactone		1	[246]	
Tricalcium phosphate-collagen-hyaluronan		1	[180]	
Poly-L-lactic acid–hydroxyapatite		1	[215]	
Collagen type I–polylactic acid		1	[217]	
Polylactic acid–polyglycolic acid with fibrin		1	[261]	
Collagen–polyglycolic acid		1	[252]	
Chondroitin sulphate C–collagen type II		1	[236]	
Fibrin hydrogel with chondroitin sulphate		1	[263]	
Chitosan-demineralised bone matrix		1	[239]	
Alginate foam-chondroitin sulphate		1	[170]	
Growth factor combined with scaffolds				
TGF-β1-loaded microspheres with chitosan microspheres		1	[262]	
TGF-β1 releasing chitosan-collagen hydrogel		1	[174]	
PEOT/PBT TGF-β1 loaded scaffolds		1	[173]	
TGF-β1-activated chitosan/gelatin		1	[249]	
PLGA nanospheres with TGF-β1		1	[172]	
TGF-β1 loaded Gelatin Microspheres		1	[175]	

##### Growth factors

The commonest used growth factors were TGF-β and the bone morphogenetic protein (BMP) family. A list of growth factors used can be seen in Table 5.

**Table 5:** Number of in vitro studies using different growth factors

Growth factor	No. of studies (%)	References	Growth factor	No. of studies (%)	References
TGF-β1	48 (44%)	[50, 169–175, 189, 190, 192, 193, 195, 199, 202, 208, 210, 211, 213, 214, 216, 217, 220, 222–224, 228, 230–232, 234, 235, 244, 246, 249, 252–256, 258, 260–263, 266, 267, 270]	SOX-5	1 (1%)	[204]
TGF-β3	32 (29%)	[51, 162, 164, 168, 177, 181–184, 197, 200, 205–207, 218, 223–225, 227, 237, 239, 240, 245, 247, 248, 250, 251, 257, 259, 267, 268, 270]	SOX-6	1 (1%)	[204]
BMP-2	13 (12%)	[188, 202, 213, 219, 225–227, 229, 264, 265, 267, 270, 271]	WNT3A	1 (1%)	[171]
FGF	9 (8%)	[171, 183, 193, 197, 198, 213, 225, 246, 258]	IL-1	1 (1%)	[197]
IGF-1	7 (6%)	[179, 184, 192, 213, 224, 254, 265]	EGF	1(1%)	[193]
BMP-6	7 (6%)	[181, 216, 219, 224, 227, 250, 266]	OP-1	1 (1%)	[222]
TGF-β2	4 (4%)	[209, 219, 238, 270]	AA2P	1 (1%)	[266]
GDF-5	3 (3%[	[48, 186, 269]	IL-10	1 (1%)	[178]
SOX-9	2 (2%)	[204, 221]	TNFα	1 (1%)	[178]
BMP-4	2 (2%)	[227, 271]	PRP	1 (1%)	[242]
DEX	2 (2%)	[224, 266]	IWP2	1 (1%)	[171]
BMP-7	1 (1%)	[219]	None	15 (14%)	[52, 176, 180, 185, 187, 191, 194, 196, 201, 212, 215, 233, 236, 241, 243]
PDGF	1 (1%)	[202]			

##### Cell seeding and passage

There was wide heterogeneity in cell seeding density and there appeared to be no standard form of measurement. Li et al. [50] examined three different seeding densities: 2, 5 and 10 × 10^6^ cells/scaffold, and found that scaffolds seeded with 5 × 10^6^ cells per scaffold induced the highest chondrogenesis; however, other groups [51–53] found that a higher seeding density results in better chondrogenesis.

Apart from 26 studies which did not state cell passage number, most studies used MSC of an early passage, anything between uncultured fresh (passage zero (P0) and five times passaged cells (P5). One study used cells of P6 [54], and another study used cells between P4 and P7 [48]. No relationship was apparent between chondrogenesis and number of passages.

##### Length of study

The length of each in vitro study can be seen in Table 6. The majority of studies were short-term models; 27 studies (25%) ended between 1 and 2 weeks, 35 studies (33%) ended between 2 and 3 weeks and 15 studies (14%) ended between 3 and 4 weeks.

**Table 6:** Length of studies

Length of study	No. of studies	References
Up to 1 week	9	[172, 203, 210, 212, 224, 229, 239, 266, 270]
1–2 weeks	27	[50, 170, 174, 178, 182, 189, 192, 194, 198, 202, 215, 218, 220, 223, 228, 234, 235, 237, 240, 249, 254, 260–265]
2–3 weeks	36	[52, 53, 168, 169, 173, 175, 179, 180, 183–186, 190, 191, 195, 196, 199, 200, 204, 205, 209, 213, 217, 225, 226, 230, 232, 233, 236, 246, 250, 256, 258, 269, 271]
3–4 weeks	15	[51, 176, 181, 188, 193, 201, 211, 216, 219, 221, 241, 251, 253, 255, 257]
4–5 weeks	7	[171, 177, 206, 214, 231, 259, 267]
5–6 weeks	10	[48, 187, 208, 222, 238, 244, 247, 248, 252, 268]
6–7 weeks	1	[207]
7–8 weeks	1	[197]
8–9 weeks	3	[164, 243, 245]
Not stated	1	[242]

##### Method of assessment

A range of techniques was used to assess chondrogenesis within the in vitro studies. These techniques consisted of histology, immunohistochemistry, qPCR, biochemical analysis, imagery and mechanical testing. The techniques used are summarised in Table 7.

**Table 7:** Types of techniques used to assess chondrogenesis of MSCs

Type of techniques	No. of studies (%)	References
Histology	87 (79%)	[48, 50–53, 164, 168–170, 173–175, 177–179, 181–187, 191–195, 197–201, 204–211, 213–217, 219–222, 226, 229, 230, 232–238, 240–248, 250, 252–264, 267–271]
Immunohistochemistry	78 (71%)	[48, 50, 52, 53, 168–171, 173–175, 178–183, 185–191, 193, 194, 197, 198, 201, 203–205, 207, 212–215, 217, 218, 220, 221, 224, 226, 228–238, 241, 242, 244, 246–248, 250–259, 264, 265, 267–271]
qPCR	70 (64%)	[53, 168, 169, 173, 174, 176, 178–186, 188, 190, 192–194, 196, 199, 200, 202–205, 207–209, 211, 214, 216–220, 222–232, 235, 236, 239, 240, 242, 246, 249–251, 256, 258, 259, 261–263, 265–267, 269–271]
Biochemical analysis	64 (58%)	[48, 50–52, 164, 168, 170–172, 176, 177, 179, 180, 182–184, 188, 189, 191, 192, 197, 199, 200, 202, 204, 205, 209, 212, 214, 216–219, 222–224, 226, 227, 233–240, 244, 245, 247–249, 252, 254, 257, 260–266, 268–270]
Imaging (confocal, SEM, TEM)	24 (22%)	[52, 172, 176, 180, 185, 187, 194, 198, 208, 215–217, 225, 226, 230, 232, 241, 242, 249, 252, 255, 262, 263, 265]
Mechanical testing	15 (14%)	[51, 52, 164, 169, 175, 193, 197, 207, 220, 245, 247, 248, 256, 257, 268]

#### Animal studies (pre-clinical)

One hundred eleven animal studies were included of which 109 were controlled laboratory studies, one was a pilot study [49] and one was a longitudinal case study on a race horse [55]. The commonest animal studied with 59 studies was rabbit (53%). The different species of animals studied is shown in Table 8.

**Table 8:** Different species of animals used to assess reparative effect of MSCs on cartilage defect

Animals	No. of studies (%)	References
Rabbits	57 (51%)	[49, 54–102, 134, 150–154, 160, 161, 207, 272–324]
Pigs	16 (14%)	[61, 62, 68–72, 87, 90, 153, 273, 276, 279, 290, 308–310]
Rats	13 (12%)	[60, 78–82, 91, 152, 160, 278, 286, 311, 312]
Sheep	8 (7%)	[89, 272, 282, 283, 313–316]
Goats	5 (5%)	[49, 95, 100, 101, 318]
Horses	4 (4%)	[55, 96, 98, 317]
Dogs	4 (4%)	[86, 97, 151, 287]
Monkeys	2 (2%)	[319, 320]
Guinea pigs	1 (<1%)	[281]
Donkeys	1 (<1%)	[57]

##### Defect

The size of the defect varied from 2 to 25 mm^2^ in the smaller animals and from 1 to 64 mm^2^ in the larger animals. All but two studies [56, 57] used the knee for defect creation.

##### Stem cell type

Bone marrow-derived stem cells were used in 84 studies (75%). Thirteen studies (11%) used adipose stem cells [54, 58–69], six (5%) used synovia [70–75] and three (2%) used periostium-derived MSCs [76–78]. Three studies (3%) used embryonic stem cell-derived MSCs [79–81] whereas 2 studies (2%) used muscle-derived MSCs [82, 83]. One group showed promising results of allogenic MSCs in a rabbit model when compared to autologous cells, although numbers were small [84, 85]. Another used compared autologous chondroprogenitor cells and allogenic chondroprogenitor cells against controls in an equine model and reported that repair tissue quality in the allogenic cell group was not superior to that in the control (fibrin only) group and also showed poorer radiographic changes in the allogenic group [23].

##### Cell culture, dose and delivery

There was much variation in the number of cells implanted and the number of cell passages from 3–10 or more [79, 86].

The number of cells varied from 4 × 10^3^ – 1 × 10^10^. The majority of studies used between 10^6^ and 10^8^ cells. Some did not specify the number of cells implanted. Two studies suggested that improved chondrogenesis occurs with a higher implanted cell number [75, 87], although others suggested that the high cell numbers increase the risk of synovitis [75] and synovial proliferation [88].

The cells were transplanted into the defect both as cell therapy (injection directly into the joint) (17 studies, 15%) or by tissue engineering (cell-scaffold combinations) (94 studies, 85%). Fifteen studies [49, 65, 72, 75, 81, 86, 89–97] used a mixture of solutions prepared from hyaluronic acid [65, 92, 94–97], phosphate buffer solution [91], plasma [75], basal medium with chondrogenesis [89], collagen acid [93], sodium alginate [86] or a growth factor medium [90]. Two studies used MSCs only [49, 72].

##### Scaffold

Ninety-two studies (82%) used a scaffold. The material used was a synthetic polymer either collagen based, fibrinogen glue or a synthetic protein (e.g. rHuBMP-2) in 62 (56%) studies (Table 9).

**Table 9:** Table showing the types of scaffold used in animal studies

Scaffold type	No. of studies	References
No Scaffold	19 (17%)	[49, 54, 61, 70, 72–75, 81, 86, 89–91, 97, 100, 102, 280–282, 284]
Poly (lactide-co-glycoside) PLGA	17 (16%)	[56, 59, 62, 63, 83, 88, 150, 153, 160, 277, 285, 286, 289–292, 316]
Fibrin/Fribrin glue	11 (9%)	[55, 64, 76–78, 152, 278, 293, 308, 317, 318]
Hydrogel	9 (8%)	[65, 69, 81, 94, 279, 288, 314, 321, 323]
Collagen	9 (8%)	[79, 80, 134, 276, 299, 301, 309, 320, 322]
Hyaluronic acid	7 (6%)	[57, 92, 95, 96, 273, 304, 324]
Alginate beads	4 (3%)	[65, 84, 101, 294]
Tissue membrane	4 (3%)	[82, 98, 303, 305]
Polyglycolic acid	3 (3%)	[99, 161, 274]
PGA/PLA	3 (3%)	[68, 290, 296]
Hylauronan crosslinked matrix	2 (2%)	[154, 297]
Poly-L-lactide-co-caprolactone	2 (2%)	[275, 300]
Polycaprolactone cartilage (PCL)	2 (2%)	[87, 272]
Animal-origin osteochondral plug scaffold	2 (2%)	[272, 298]
Chitosan microspheres and fibrin glue	1 (<1%)	[60]
Gel carries (collagen/HA/Fibrogen)	1 (<1%)	[71]
Polychoxanone/poly(vinyl alcholo) PDO/PVA	1 (<1%)	[302]
Cartilage aggregate	1 (<1%)	[306]
Collagen/glycosaminoglycan porous titanium biphasic scaffold	1 (<1%)	[151]
Articular chondrocyte seeded matrix associated autologous chondrocyte transplant (MACT)	1 (<1%)	[313]
MSC-ADM (accellulo-dermal matrix)	1 (<1%)	[319]
Hyaff-11 scaffold	1 (<1%)	[295]
Porous-gelatin-chonroitin hyaluronate	1 (<1%)	[291]
Bone protein 7 PCL	1 (<1%)	[66]
Human acellular amniotic membrane	1 (<1%)	[307]
Pluronic-F 127	1 (<1%)	[102]
Tricalcium phosphate	1 (<1%)	[315]
Agarose	1 (<1%)	[311]
GCH-GCBB	1 (<1%)	[93]
ACHMS (atelocollagen honeycomb-shaped membrane)	1 (<1%)	[58]
Magnet	1 (<1%)	[310]
Human cartilage extra cellular matrix 3D porous acellular	1 (<1%)	[67]

##### Growth factors

Thirty-two studies (29%) assessed the effect of growth factors on MSC chondrogenesis. Seventeen out of 38 (44%) used TGF-β1/3 (Table 10), the majority of which show a positive effect on chondrogenesis.

**Table 10:** Table showing growth factors used in animal studies

Growth factor	No. of studies	References
TGF-β3/1/2	17 (15%)	[56, 65, 66, 70, 76, 85, 90, 100, 280, 282, 285, 287, 290, 291, 309, 311, 323]
CDMP–1	2 (2%)	[56, 134]
FGF-2	2 (2%)	[90, 304]
Ad-hTGF-B1	1 (<1%)	[321]
AdBMP–2	1 (<1%)	[78]
chABC	1 (<1%)	[74]
PRP	1 (<1%)	[75]
Gene modified MSCs (gene modification to BcL-xL gene)	1 (<1%)	[299]
hiGF-1-DNA	1 (<1%)	[101]
AdIGF–1	1 (<1%)	[78]
rHuBMP–2	1 (<1%)	[82]
Ham-F-12	1 (<1%)	[303]
NaO11	1 (<1%)	[277]
NSC23766-Rac1 inhibitor	1 (<1%)	[60]

##### Associated procedures

Ten of the studies compared MSC treatment against other surgical modalities such as debridement [55], microfracture [49, 91, 96, 98, 99] and mosaicplasty [77, 100–102].

##### Outcome measures

There were a variety of outcome measures used to analyse the results of the studies. The majority of studies (79%) used evidence of hyaline-like cartilage as being a positive outcome (Tables 11 and 12).

**Table 11:** Outcome measures used in animal studies (some studies used more than one outcome measure)

Outcome score	No. of studies using the score (%)	References
Histology scores	111 (100%)	[49, 54–102, 134, 150–154, 160, 161, 272–324]
International Cartilage Repair Society Score	26 (23%)	[49, 60, 61, 63, 66, 69, 72, 74, 79, 89, 92, 94, 98, 99, 272, 282, 283, 289, 305, 306, 310, 313, 314, 316, 319, 324]
Wakitani score	21 (19%)	[58, 62, 67, 68, 72, 73, 80, 82, 97, 151, 273, 277, 279, 284, 285, 290, 299, 304, 310, 321]
O’Driscoll score	2018%	[49, 71, 81, 84, 85, 93, 100, 160, 272, 276, 290, 296–298, 302, 306, 308, 313, 314, 322]
Functional scores/mechanical	11 (10%)	[55, 57, 62, 67, 69, 81, 101, 277, 287, 290, 315]
MRI scores	5 (5%)	[63, 69, 96, 101, 316]
Arthroscopy scores	5 (5%)	[72, 96, 310, 317, 318]
Macroscopic osteoarthritis score	3 (3%)	[57, 281, 295]
Pineda score	3 (3%)	[290, 293, 309]
Schreiber score	2 (2%)	[101, 300]
Britternberg score	2 (2%)	[84, 85]
Slochagg score	1 (<1%)	[300]
Moran score	1 (<1%)	[64]
Gill score	1 (<1%)	[95]

**Table 12:** Analysis technique used on repaired tissue

Analysis used	No. of studies (%)	References
Hyaline-like cartilage	88 (79%)	[49, 54–56, 58, 59, 61, 62, 64–69, 71–73, 75, 76, 78–89, 92, 95, 97, 98, 100, 101, 134, 150–152, 154, 160, 161, 273–280, 285–302, 304, 305, 307, 309, 310, 312, 314–324]
Collagen type II	84 (76%)	[54, 56–59, 62, 65–73, 75–88, 90, 91, 93–96, 98, 100–102, 134, 150–154, 160, 161, 272–276, 278–282, 284–288, 292, 294–296, 300, 302–306, 308, 309, 311, 313–315, 317–319, 321, 323]
Cluster Chondrocytes	34 (31%)	[57, 60, 62, 63, 72, 74, 77, 78, 80, 81, 83, 84, 91, 97, 102, 151, 152, 160, 161, 273, 276, 280, 281, 283, 291, 292, 296, 297, 304, 312, 318, 319, 322, 324]
Glycosaminoglycan	40 (36%)	[49, 62, 65, 67–71, 73–75, 81, 85, 87, 94, 96–101, 160, 272, 274, 279, 282, 286, 288, 290, 291, 296, 300, 301, 308, 309, 311, 312, 315, 319, 323]
Genes	22 (20%)	[56, 60, 61, 63, 64, 66, 78, 80, 82, 90, 94, 96, 134, 275, 277, 283, 285, 294, 311, 316, 321, 323]
Proteoglycan	8 (7%)	[56, 63, 84, 95, 98, 287, 294, 295]

#### Human studies (clinical)

Thirty-one published studies by 15 different groups looked at clinical applications of MSCs. One used allogenic stem cells [103] and the rest autologous stem cells. The types of studies can be seen in Tables 13 and 14.

**Table 13:** Number of publications for each study type and phase

Category	No. of studies (total 28)	References
Phases of clinical studies		
Pilot/feasibility study incl. case report	15 (54%)	[104–108, 118, 119, 122, 124–129, 133]
Phase 1 (safety assessment)	8 (26%)	[109–112, 116, 123, 130, 131]
Phase 2 (efficacy assessment)	8 (26%)	[103, 113–115, 117, 120, 121, 132]
Phase 3 (large scale efficacy assessment through a multi-centre RCT)	0 (0%)	–
Phase 4 (post-market surveillance)	0 (0%)	–

**Table 14:** Summary of the published clinical studies

Category	No. of studies	References
Cell source		
Bone marrow	22 (71%)	[103–105, 109, 111–113, 115–118, 120, 122–128, 130–132]
Adipose	5 (16%)	[106–108, 110, 114]
Peripheral blood	2 (6%)	[119, 121]
Synovium	2 (6%)	[129, 133]
Cell delivery		
Arthroscopic implantation		
Hyaluronic acid membrane	2 (6%)	[117, 130]
Hyaluronic acid with fibrin glue or platelet gel	2 (6%)	[116, 128]
Polyglycolic acid/hyaluronan	2 (6%)	[127, 131]
Collagen with platelet gel	1 (3%)	[116]
Fibrin glue	1 (3%)	[108]
HYAFF 11 scaffold	1 (3%)	[132]
Acetate Ringer solution	1 (3%)	[133]
Unspecified	1 (3%)	[107]
Intra-articular injection		
PBS only	2 (6%)	[104, 110]
PBS with HA	2 (6%)	[119, 121]
Autologous serum	2 (6%)	[115, 123]
Ringer lactate solution	3 (10%)	[103, 111, 112]
PBS with serum albumin	1 (3%)	[105]
HA and PRP	1 (3%)	[106]
PRP	1 (3%)	[114]
Commercial serum	1 (3%)	[109]
Transplantation by open surgery		
Collagen	6 (21%)	[103, 113, 118, 122, 124, 126, 129]
Ascorbic acid-mediated sheet	2 (7%)	[120, 123]
Fibrin glue	1 (4%)	[125]
Cell dose		
Less than 10 million	8 (26%)	[105, 107, 108, 114, 120, 122, 124, 129]
10–20 million	5 (16%)	[113, 118, 119, 123, 125]
Over 20 million	7 (23%)	[103, 104, 109–112, 133]
Unspecified	11 (35%)	[106, 115–117, 121, 126–128, 130–132]
Follow-up		
Up to 6 months	4 (13%)	[104–106, 110]
Up to 12 months	6 (19%)	[103, 109, 111, 124, 125, 127]
Up to 2 years	11 (35%)	[107, 113–116, 120, 121, 128–131]
Up to 3 years	7 (23%)	[108, 112, 117, 119, 122, 126, 132]
Over 3 years	2 (6%)	[118, 133]
Assessments		
Radiology (MRI, X-ray)	24 (77%)	[103–106, 109–112, 115–117, 119, 121–125, 127–133]
Arthroscopic assessment incl. histology	17 (54%)	[107, 108, 113, 116–122, 124–126, 130–133]
IKDC	10 (32%)	[107, 108, 115, 121, 122, 126, 128, 130–132]
VAS pain	12 (39%)	[103–106, 109–112, 114, 129, 131, 132]
Tegner activity scale	8 (26%)	[107, 108, 114, 115, 129, 131–133]
Lysholm	6 (19%)	[114, 115, 125, 128, 131, 133]
KOOS	5 (16%)	[126, 128–130, 132]
Function (no scoring systems or unspecified)	4 (13%)	[104–106, 109]
ICRS cartilage injury evaluation package	3 (10%)	[120, 123, 125]
Clinical symptoms/outcomes (no scoring system or unspecified)	3 (10%)	[105, 109, 124]
(Revised) Hospital for special surgery knee-rating scale	2 (6%)	[113, 125]
Functional Rating Index	2 (6%)	[104, 106]
WOMAC	5 (16%)	[103, 109–112]
AOFAS score	2 (6%)	[112, 116, 117]
Knee Society Score	1 (3%)	[110]
Harris Hip Score	1 (3%)	[112]
Concomitant procedures		
Subchondral bone marrow stimulation (multiple perforation, drilling, abrasion chondroplasty)	11 (35%)	[113, 115, 118, 119, 121–123, 125, 127, 128, 131]
Debridement, synovectomy, excision of degenerative tears (no subchondral bone marrow stimulation)	8 (26%)	[107, 108, 114, 116, 117, 124, 130, 133]
ACL reconstruction, meniscus repair, osteotomy, or patella alignment, ACL calcification removal, trochlear resurfacing, osteochondral fragment fixation	8 (26%)	[115, 123, 126, 129–133]
None	6 (19%)	[103, 105, 106, 110–112]
Not specified	3 (10%)	[104, 109, 120]
Previous procedures		
Microfractures/multiple perforation/multiple drilling	6 (19%)	[104, 116, 117, 122, 125, 130]
Menisectomy	6 (19%)	[103, 111, 124, 129, 131, 133]
ACL reconstruction	4 (13%)	[103, 111, 131, 133]
Multiple (microfracture, debridement)	1 (3%)	[119]
ACI	2 (6%)	[116, 117]
None	6 (19%)	[106–108, 110, 114, 118]
Not specified	9 (29%)	[105, 109, 112, 115, 120, 121, 126, 128, 132]

*PBS* phosphate-buffered saline, *HA* hyaluronic acid, *PRP* plate-rich-plasma, *RCT* randomised controlled study, *KOOS* Knee and Osteoarthritis Outcome Score, *IKDC score* International Knee Documentation Committee Score, *WOMAC* the Western Ontario and McMaster Universities Arthritis Index, *AOFAS* the American Orthopaedic Foot & Ankle Society

There were 52 unpublished clinical trials, majority of which are early phase studies (I–II; 63%) and only 5 trials were phase II/III. Table 15 shows a summary of these clinical trials.

**Table 15:** Clinical trials (unpublished/on-going) registered in ClinicalTrials.gov

Title	Cell source	Country	Clinical trial phase	Condition	Study design	Enrolment	Follow-up	Arm(s)	Cell delivery	Primary outcomes	Study status (on 8.3.2016)	ClinicalTrials.gov Identifier
Autologous cells												
Mesenchymal Stem Cells in Knee Cartilage Injuries	Bone marrow	Jordan	II	Advanced knee articular cartilage injury	Non-randomized parallel assignment; double blind	13	12 months	Culture expanded MSCs alone vs. MSC with platelet lysate	Intra-articular injection	Therapeutic benefit	Completed in August 2015; no publication found	NCT02118519
Adult Stem Cell Therapy for Repairing Articular Cartilage in Gonarthrosis	Bone marrow	Spain	I/II	Gonarthrosis grade 2–3	Open label; single group assignment	15	12 months	Culture expanded MSCs (40 million cells)	Articular injection	Feasibility/safety	Completed in January 2013; no publication found	NCT01227694
Autologous Bone Marrow Mesenchymal Stem Cells Transplantation for Articular Cartilage Defects Repair	Bone marrow	UK	I/II	Knee articular cartilage defects	Randomized parallel assignment; double blind	10	12 months	MSCs (fresh or cultured unspecified)	Intra-articular injection	Change in WOMAC	Unknown (estimated study completion date; July 2014)	NCT01895413
Mesenchymal Stem Cell for Osteonecrosis of the Femoral Head	Bone marrow	China	0	Osteochondritis of the femoral head	Open label single group assignment	15	5 years	Culture expanded MSC and bone marrow nuclear cells	Infusion through medial femoral circumflex artery, lateral femoral circumflex artery and obturator artery	Femoral head blood-supply artery angiographies; femoral head necrosis	Unknown (estimated study completion date; August 2015)	NCT00813267
The Effects of Intra-articular Injection of Mesenchymal Stem Cells in Knee Joint Osteoarthritis	Bone marrow	Iran	II	Knee joint osteoarthritis	Single centre, randomised, placebo controlled, double blind	40	3 months	Culture-expanded MSCs vs. placebo	Intra-articular injection	Changes in WOMAC physical function and VAS pain	Completed in November 2012; no publication found	NCT01504464
Safety and Efficacy of Autologous Bone Marrow Stem Cells for Treating Osteoarthritis	Bone marrow	India	I/II	Knee OA Kellgren and Lawrence classification 3–4	Open label single group assignment; multi-centre	10	1 year	MSCs (fresh or culture-expanded unspecified)	Unknown	WOMAC pain score and safety	On-going (estimated study completion date; January 2012)	NCT01152125
Treatment of Knee Osteoarthritis by Intra-articular Injection of Bone Marrow Mesenchymal Stem Cells	Bone marrow	Spain	I/II	Knee OA	Randomised parallel assignment; open label	30	12 months	Culture-expanded MSCs (10 million or 100 million cells) and hyaluronic acid (HyalOne®) vs. HyalOne®	Intra-articular injection	Pain and function (VAS, WOMAC, KOOS, EuroQol, SF-16, Lequesne), radiographic	On-going (estimated study completion date; February 2015)	NCT02123368
Intra-Articular Autologous Bone Marrow Mesenchymal Stem Cells Transplantation to Treat Mild to Moderate Osteoarthritis	Bone marrow	Malaysia	II	Mild to moderate OA based on Kellgren-Lawrence radiographic classification	Randomised parallel assignment; open label	50	12 months	MSCs (fresh or culture-expanded unspecified) in hyaluronic acid “Orthovisc” vs. hyaluronic acid	Intra-articular implantation	Changes in cartilage thickness (MRI)	Unknown (estimated study completion date; March 2014)	NCT01459640
Treatment of Osteoarthritis by Intra-articular Injection of Bone Marrow Mesenchymal Stem Cells With Platelet Rich Plasma (CMM-PRGF/ART)	Bone marrow	Spain	I/II	Knee OA	Randomised parallel assignment; open label; multi-centre	38	12 months	Culture-expanded MSCs with PRP (PRGF®) vs. PRGF® only	Intra-articular injection	Pain and function (VAS, WOMAC, KOOS, EuroQol, SF-16, Lequesne), radiographic	On-going (estimated study completion date; June 2017)	NCT02365142
Mesenchymal Stem Cells Enhanced With PRP Versus PRP In OA Knee (MSCPRPOAK)	Bone marrow	India	I/II	Knee OA grade 1–2 Ahlbacks radiographic staging	Randomised parallel assignment double blinded	24	6 months	Culture-expanded MSCs (10 million cells) with autologous PRP vs. PRP only	Injected by lateral approach	VAS pain	Unknown (estimated study completion date; June 2014)	NCT01985633
Side Effects of Autologous Mesenchymal Stem Cell Transplantation in Ankle Joint Osteoarthritis	Bone marrow	Iran	I	Severe ankle OA	Single group assignment open label	6	6 months	Culture-expanded MSCs	Intra-articular injection	Safety	Completed in September 2011; no publication found	NCT01436058
Human Autologous MSCs for the Treatment of Mid to Late Stage Knee OA	Bone marrow	Canada	I/II	Mid- to late-stage knee OA	Single group assignment, open label	12	1 year	Culture-expanded MSCs (1 million, 10 million or 50 million cells)	Injection	Safety	On-going (estimated study completion date; February 2021)	NCT02351011
A Controlled Surveillance of the Osteoarthritic Knee Microenvironment With Regenexx® SD Treatment	Bone marrow	USA	NA	Knee OA Kellgren-Lawrence grade 2 or greater	Observational cohort study	20	6 weeks	Regenexx® SD (bone marrow concentrate)	Injection	Temporal median change in protein concentration or percentage of cellular subpopulations	On-going (estimated study completion date; March 2016)	NCT02370823
The Effect of Platelet-rich Plasma in Patients With Osteoarthritis of the Knee	Bone marrow	Iran	III	Knee OA grade 2 and above (radiographic)	Randomised, parallel assignment, placebo controlled, double blinded	50	2 year	Bone marrow aspirate vs. placebo (saline)	Intra-articular injection	VAS pain, WOMAC physical activity, cartilage repair (MRI)	Completed in April 2014; no publication found	NCT02582489
Outcomes Data of Bone Marrow Stem Cells to Treat Hip and Knee Osteoarthritis	Bone marrow	USA	NA	Hip and knee OA	Observational cohort study	12	1 year	Bone marrow concentrate	Injection	VAS pain, Harris Hip Score or Knee Society Score, Physician Global Assessment	Completed in March 2014; no publication found	NCT01601951
Use of Autologous Bone Marrow Aspirate Concentrate in Painful Knee Osteoarthritis (BMAC)	Bone marrow	USA	II	Bilateral knee OA Kellgren-Lawrence grade 1–3	Randomised, parallel assignment, placebo controlled, single blinded	25	12 months	Bone marrow concentrate vs. placebo (saline)	Injection	Safety	On-going (estimated study completion date; December 2016)	NCT01931007
Autologous Stem Cells in Osteoarthritis	Bone marrow	Mexico	I	Knee OA Kellgren-Lawrence radiographic scale grade 2–3	Randomised parallel assignment, open label	61	6 months	Hematopoietic stem cells (fresh) vs. acetaminophen (750 mg orally TID)	Infusion	Safety	Completed in May 2014; no publication found	NCT01485198
The Use of Autologous Bone Marrow Mesenchymal Stem Cells in the Treatment of Articular Cartilage Defects	Bone marrow	Egypt	Not given	An isolated osteochondral defect with no more than grade 1 or 2 Outerbridge	Single group assignment, open label	25	12 months	Culture-expanded MSCs	Open surgery or arthroscopy	Clinical scores and radiological images	Unknown (estimated study completion date; December 2014)	NCT00891501
Autologous Transplantation of Mesenchymal Stem Cells (MSCs) and Scaffold in Full-thickness Articular Cartilage	Bone marrow	Iran	I	Full-thickness chondral defects	Single group assignment, open label	6	12 months	Culture-expanded MSCs mixed with collagen I scaffold	Unspecified	Knee cartilage defects	Completed in December 2010; no publication found	NCT00850187
“One-step” Bone Marrow Mononuclear Cell Transplantation in Talar Osteochondral Lesions (BMDC)	Bone marrow	USA	III	ICRS grade 3–4 Osteochondral lesions of the talar dome	Single group assignment, open label	140	24 months	Bone marrow concentrate	Arthroscopy	American Orthopaedic Foot and Ankle Society hindfoot score	On-going (estimated completion date; June 2016)	NCT02005861
Transplantation of Bone Marrow Stem Cells Stimulated by Proteins Scaffold to Heal Defects Articular Cartilage of the Knee	Bone marrow	France	0	Knee OA ICRS classification grade 4	Single group assignment, open label	50	1 year	Freshly isolated bone marrow mononuclear cells mixed with protein scaffold	Arthroscopy (one step procedure)	IKS	Unknown (estimated completion date; December 2014))	NCT01159899
INSTRUCT for Repair of Knee Cartilage Defects	Bone marrow	The Netherlands	Not given	Knee articular cartilage defect	Single group assignment, open label; multi-centre	40	1 year	INSTRUCT scaffold (biodegradable scaffold seeded with autologous primary chondrocytes and bone marrow cells)	Arthrotomy	Safety and lesion filling	Completed in June 2014; no publication found	NCT01041885
HyaloFAST Trial for Repair of Articular Cartilage in the Knee (FastTRACK)	Bone marrow	Hungary	Not given	Knee articular cartilage defect	Randomised, parallel assignment, placebo controlled, single blinded, multi-centre	200	2 years	Hyalofast® scaffold with bone marrow aspirate concentrate vs. microfracture	One-step arthroscopic procedure	Changes in KOOS	On-going (estimated study completion date; June 2020)	NCT02659215
Autologous Adipose Stem Cells and Platelet Rich Plasma Therapy for Patients With Knee Osteoarthritis	Adipose	Vietnam	I/II	Idiopathic or secondary knee OA grade 2–3 radiographic severity	non-randomised unblinded	16	12 months	Stromal vascular fraction (10–50 million cells) and platelet rich plasma (PRP)	Injection	Safety	Completed in December 2015; no publication found	NCT02142842
Effectiveness and Safety of Autologous ADRC for Treatment of Degenerative Damage of Knee Articular Cartilage	Adipose	Russia	I/II	Knee OA (degenerative damage of knee articular cartilage)	Single group assignment, open label	12	24 weeks	Adipose-derived regenerative cells (ADRC) extracted using Celution 800/CRS System (Cytori Therapeutics, Inc.)	Intra-articular injection	Safety	On-going (estimated study completion date; December 2016)	NCT02219113
Autologous Adipose-Derived Stromal Cells Delivered Intra-articularly in Patients With Osteoarthritis	Adipose	USA	I/II	OA	Single group assignment, open label, multi-centre	500	6 months	MSCs in PRP	Intra-articular injection	Pain score, functional rating index, visual analogue scale (VAS), physical therapy (PT) and range of motion (53), quality of life scores, reduction in analgesics, adverse events	On-going (estimated study completion date; December 2016)	NCT01739504
Mesenchymal Stem Cell Treatment for Primary Osteoarthritis Knee	Adipose	Taiwan	I	Bilateral primary OA Kellgren and Lawrence grade 2–3 as determined by X-ray	Single group assignment, open label,	10	12 months	MSCs (8–10 million cells)	Intra-articular injections	Safety	On-going (estimated study completion date; December 2016)	NCT02544802
Autologous Adipose Tissue-Derived Mesenchymal Progenitor Cells Therapy for Patients With Knee Osteoarthritis	Adipose	China	II	Knee OA	Single group assignment, double blinded	48	6 months	Fresh MSCs (10 million, 20 million, 50 million cells twice) vs. placebo (PBS)	Intra-articular injection	WOMAC score	Completed in December 2013; no publication found	NCT01809769
Clinical Trial of Autologous Adipose Tissue-Derived Mesenchymal Progenitor Cells (MPCs) Therapy for Knee Osteoarthritis	Adipose	China	II	Knee OA	Randomised, parallel assignment, placebo controlled, single blinded	48	12 months	Culture-expanded MSCs vs. sodium hyaluronate	Intra-articular injection	WOMAC	On-going (estimated study completion date; July 2016)	NCT02162693
Outcomes Data of Adipose Stem Cells to Treat Osteoarthritis	Adipose	USA	NA	Knee OA	Observational cohort study	50	12 months	Cellular concentrate	Unknown	KOOS, HOOS	On-going (estimated study completion date; September 2017)	NCT02241408
Clinical Trial to Evaluate Efficacy and Safety of JOINTSTEM in Patients With Degenerative Arthritis	Adipose	Korea	II/III	Knee OA	Randomised parallel assignment, double blinded	120	24 weeks	MSCs (100 million cells) vs. sodium chloride	Injection	WOMAC	On-going (estimated study completion date; July 2017)	NCT02658344
ADIPOA–Clinical Study	Adipose	France	I	Moderate or severe knee OA	Non-randomised parallel assignment, open label	12	1 year	MSCs (2 million, 10 million, 50 million cells)	Intra-articular injection	Safety	Completed in December 2014; no publication found	NCT01585857
Safety and Clinical Effectiveness of A3 SVF in Osteoarthritis	Adipose	USA	Not given	OA	Single group assignment, open label	30	1 year	Stromal vascular fraction with activated platelet	Injection	Pain and inflammation–WOMAC scores, comprehensive inflammation blood panel	On-going (estimated study completion date; September 2015)	NCT01947348
Safety and Clinical Outcomes Study: SVF Deployment for Orthopaedic, Neurologic, Urologic, and Cardio-pulmonary Conditions	Adipose	USA	Not given	Neurodegenerative diseases, OA, erectile dysfunction, autoimmune diseases, cardiomyopathies or emphysema	Single group assignment, open label	3000	36 months	Stromal vascular fraction	Intra-venous, intra-articular, and soft tissue injection	Safety	On-going (estimated study completion date; March 2018)	NCT01953523
Microfracture Versus Adipose-Derived Stem Cells for the Treatment of Articular Cartilage Defects	Adipose	USA	Not given	Knee OA	Randomised, parallel assignment, double blind	90	24 months	Fibrin glue + acellular collagen dermal matrix + DSCs, + additional layer of fibrin glue vs. microfracture	Arthroscopy	KOOS	On-going (estimated study completion date; December 2020)	NCT02090140
Autologous Mesenchymal Stem Cells vs. Chondrocytes for the Repair of Chondral Knee Defects (ASCROD)	Adipose	Spain	I/II	Articular cartilage lesion of the femoral condyle	Randomised, parallel assignment, open label	30	18 months	Cultured stem cells vs. cultured autologous chondrocytes	Unknown	Hyaline cartilage production for chondral knee lesions repair	Unknown (estimated study completion date; June 2012)	NCT01399749
A Phase 2 Study to Evaluate the Efficacy and Safety of JointStem in Treatment of Osteoarthritis	Adipose	USA	II	Knee OA	Randomised, parallel assignment, double blinded	45	6 months	Joint stem adipose-derived (MSCs) vs. Synvisc-One (hyaluronic acid)		Cartilage volume, cartilage articular surface area, cartilage thickness, subchondral bone surface curvature (MRI)	On-going (estimated study completion date; September 2017)	NCT02674399
Allogenic cells												
Treatment of Knee Osteoarthritis With Allogenic Mesenchymal Stem Cells (MSV_allo)	Bone marrow	Spain	I/II	Knee OA grade 2–4 of Kellgren and Lawrence	Randomised, parallel assignment, double blinded	30	1 years	Culture-expanded MSCs (40 million cells) vs. hyaluronic acid	Intra-articular transplantation	Safety	Completed in June 2014; published in August 2015	NCT01586312 (Linked to study NCT01183728)
Clinical Trial of Allogenic Adipose Tissue-Derived Mesenchymal Progenitor Cells Therapy for Knee Osteoarthritis	Adipose	China	I	Degenerative arthritis by radiographic criteria of Kellgren Lawrence	Randomised, parallel assignment, double blind	18	48 weeks	10 million MSCs vs. 20 million MSCs	Intra-articular injection	WOMAC	On-going (estimated study completion date; July 2017)	NCT02641860
Clinical Study of Umbilical Cord Tissue Mesenchymal Stem Cells (UC-MSC) for Treatment of Osteoarthritis	Umbilical Cord	Panama	I/II	Modified Kellgren-Lawrence classification grade 2–4 radiographic OA severity.	Randomised, parallel assignment, open label	40	12 months	Single intra-articular injection of MSCs vs. IV injections of MSC for 3 days	Intra-articular injection; IV	Safety	On-going (estimated study completion date; March 2017)	NCT02237846
Safety and Feasibility Study of Mesenchymal Trophic Factor (MTF) for Treatment of Osteoarthritis	Umbilical Cord	Panama	I/II	Modified Kellgren-Lawrence classification grade 2–4 radiographic OA severity.	Non-Randomised, single group assignment, open label	40	12 months	Intra-articular injection of allogeneic MTF from UC-MSC vs. 12 subcutaneous MTF injections, once per week	Intra-articular injection; subcutaneous injection	Safety	On-going (estimated study completion date; June 2017)	NCT02003131
A Study to Assess Safety and Efficacy of Umbilical Cord-derived Mesenchymal Stromal Cells in Knee Osteoarthritis	Umbilical Cord	Chile	I/II	Kellgren-Lawrence classification grade 1–3 radiographic OA severity	Randomised, parallel assignment, double blind	30	12 months	MSCs (single dose of 20 million MSCs or double dose at 6 month interval) vs. hyaluronic acid	Intra-articular injection	Safety	On-going (estimated study completion date; December 2016)	NCT02580695
Human Umbilical Cord Mesenchymal Stem Cell Transplantation in Articular Cartilage Defect	Umbilical Cord	China	I	Kellgren-Lawrence classification grade 2–4 radiographic OA severity	Single group assignment, open label	20	12 months	20 million cells every month for 4 months	Intra-articular injection	Safety	On-going (estimated study completion date; December 2016)	NCT02291926
Evaluation of Safety and Exploratory Efficacy of CARTISTEM®, a Cell Therapy Product for Articular Cartilage Defects	Umbilical cord blood	Korea	I/II	Focal, full-thickness grade 3–4 articular cartilage defects	Single group assignment, open label	12	12 months	CARTISTEM® (cultured UC MSCs mixed with sodium hyaluronate)	Unknown	Safety	On-going (estimated study completion date; May 2017)	NCT01733186
Study to Compare the Efficacy and Safety of Cartistem® and Microfracture in Patients With Knee Articular Cartilage Injury or Defect	Umbilical cord blood	Korea	III	Knee Articular Cartilage Injury or Defect	Randomised, parallel assignment, open label	104	48 weeks	CARTISTEM® (cultured UC MSCs mixed with sodium hyaluronate) vs. Microfracture	Surgery	CRS cartilage repair assessment	Completed in January 2011; no publication found	NCT01041001
Follow-Up Study of CARTISTEM® vs. Microfracture for the Treatment of Knee Articular Cartilage Injury or Defect	Umbilical cord blood	Korea	III	Knee articular cartilage injury or defect	Randomised, parallel assignment, open label	103	60 months	CARTISTEM® (cultured UC MSCs mixed with sodium hyaluronate) vs. microfracture	Unknown	IKDC, VAS pain, WOMAC	On-going (estimated study completion date; May 2015)	NCT01626677
Injections of FloGraft Therapy, Autologous Stem Cells, or Platelet Rich Plasma for the Treatment of Degenerative Joint Pain	Amniotic fluid	USA	NA	Pain associated with one of the following conditions: lumbar facet degeneration, degenerative condition causing upper extremity joint pain or degenerative condition causing lower extremity joint pain	Cohort observational study	300	24 weeks	FloGraft^TM^ (allogenic amniotic fluid-derived allograft) vs. autologous BMMSCs vs. platelet rich plasma	Injection	Pain	On-going (estimated study completion date; June 2016)	NCT01978639
IMPACT: Safety and Feasibility of a Single-stage Procedure for Focal Cartilage Lesions of the Knee	Unspecified	The Netherlands	I/II	Full-thickness articular cartilage lesion on the femoral condyle or trochlea	Single-group assignment, open label	35	18 months	Autologous chondrons (chondrocytes with their pericellular matrix) and allogeneic MSCs in the fibrin glue carrier	Unspecified (single stage surgery)	Safety	On-going (Estimated Study Completion Date: August 2015)	NCT02037204
Allogeneic Mesenchymal Stem Cells in Osteoarthritis	Unspecified	India	II	Kellgren and Lawrence classification grade 2–3 radiographic OA severity	Randomised, double blind, multi-centre	60	2 years	Culture-expanded MSCs in 2 ml plasmalyte + 2 ml, hyaluronan vs. 2 ml, plasmalyte + 2 ml, hyaluronan	Intra-articular	Safety and tolerability	Unknown (estimated study completion date; July 2014	NCT01453738
Allogeneic Mesenchymal Stem Cells for Osteoarthritis	Unspecified	Malaysia	II	Kellgren and Lawrence classification grade 2–3 OA	Randomised, double blind, multi-centre	72	1 year	Culture-expanded MSCs in 2 ml plasmalyte + 2 ml, hyaluronan vs. 2 ml, plasmalyte + 2 ml, hyaluronan	Intra-articular	Safety and tolerability	Unknown (estimated study completion date; February 2013)	NCT01448434
Autologous or allogenic unspecified												
Transplantation of Bone Marrow Derived Mesenchymal Stem Cells in Affected Knee Osteoarthritis by Rheumatoid Arthritis	Bone marrow	II/III	Iran	Rheumatoid arthritis	Randomised, parallel assignment, open label	60	6 months	MSCs vs. saline	Intra-articular injection	Pain	Completed in December 2011; no publication found	NCT01873625
Safety and Efficacy Study of MSB-CAR001 in Subjects 6 Weeks Post an Anterior Cruciate Ligament Reconstruction	Unknown	I/II	Australia	Anterior cruciate ligament injury	Randomised, parallel assignment, double blind	24	2 year	MSB-CAR001 (a preparation of MSCs) with hyaluronan vs. hyaluronan alone	Injection	Safety	Unknown	NCT01088191

##### Defects

The majority of studies (42%) used MSCs to treat knee osteoarthritis [103–115]. The rest of the studies looked at knee cartilage defects except for two which studied the ankle talar dome [116, 117]. One study used MSCs to treat knee osteoarthritis (OA), knee OA and ankle OA [112].

Of the knee cartilage defects, the patients were heterogeneous with varying defect sizes and locations, including the patellae [118–121], patella-femoral joints [122, 123], femoral condyle [113, 119–121, 123–132], trochlear [119–121] and tibial plateau [121]; and several had multiple defect sites [105, 120, 123, 128].

##### Previous treatment and associated procedures

The majority of patients who received MSC treatment had undergone previous arthroscopy [103, 104, 118, 119, 122, 124, 130], failed debridement [113, 118, 119, 121–123, 125, 127, 131] or bone marrow stimulation [114, 116, 117, 126].

##### Cell harvest source

Twenty-one studies (68%) used bone marrow-derived MSCs from the anterior or posterior superior iliac spine [103–105, 109, 111–113, 115–118, 120, 122–128, 130–132]. Five studies (18%) used adipose-derived MSCs [106–108, 110, 114], two studies (7%) used synovium-derived MSCs [129, 133] and two studies (7%) used peripheral blood progenitor cells collected by apheresis [119, 121].

##### Cell stage

Twenty studies (61%) culture-expanded their cells [103–105, 107–113, 115, 118, 120, 122–126, 129, 133], whereas 11 studies (39%) used fresh concentrated stem cells from bone marrow [116, 117, 127, 128, 130–132], fat tissues [106, 114] or peripheral blood [119, 121] in a one stage-procedure. In studies using bone marrow concentrate, approximately 60 ml of bone marrow aspirate was harvested and concentrated down to a volume of 2–4 ml before use [116, 117, 127, 130–132]. In studies using culture-expanded cells, the majority used cells from early passages, P1–P3 [103, 105, 109, 110, 112, 113, 115, 118, 120, 122–125, 129]. One study reported the use of cells at a late passage (P5) [104] ,and five studies did not specify a passage number [107, 108, 111, 126, 133].

Thirteen studies (42%) confirmed the phenotype of cells before clinical application [105, 108–110, 112, 115, 119, 120, 122–125, 129]. Commonly used surface markers to select MSCs were CD29, CD44, CD73, CD90 and CD105. Also CD14, CD34 and HLA-DR were used to eliminate non-MSCs.

##### Cell dose and delivery

The number of cells applied (dose) varied from 2–57 million for bone marrow-derived MSCs [103–105, 109, 111–113, 118, 120, 122–125, 129] and from 1.2–100 million for adipose-derived MSCs [107, 108, 110, 114]. For synovial MSCs, 8–77 million cells were used [129, 133], and for peripheral blood progenitor cells, 20 million cells were used [119]. Also, the methods for implantation varied from arthroscopic implantation (35%) [107, 108, 116, 117, 127, 128, 130–133], intra-articular injection [103–106, 109–112, 114, 115, 119, 121, 123] or open surgery (29%) [113, 118, 120, 122–126, 129].

In the cell therapy studies, the cells were suspended with a variety of different co-stimulators, including hydroxyapatite (HA) [106, 119, 121, 123], platelet rich plasma (PRP) [106, 114] and platelet lysate [104]. Some studies also administered multiple injections of stem cells [119, 121] and/or further injection of HA [115, 119, 121, 123], PRP [106, 114] or nucleated cells [104] following a stem cell injection.

The most frequently used scaffolds were type I collagen of porcine or bovine origin [113, 118, 122, 124, 126, 129], followed by ascorbic acid sheet [120, 123] and platelet-rich fibrin glue mixture [108, 125].

##### Rehabilitation

Early continuous passive motion was employed in 14 studies [113, 117–122, 124–127, 129–131]. Six studies did not report details on post-operation rehabilitation [104–106, 109, 116, 132]. Three studies aimed for full weight bearing very early by week 4 [107, 108, 122] whereas 11 studies (40%) aimed for full weight bearing by the 6th–8th week [113, 117–121, 124, 125, 127, 131, 133]. No study addressed the effect of rehabilitation on the quality of the repair.

##### Outcomes

Most commonly used outcome measures for treatment efficacy were radiological (77%) [103–106, 109–112, 115–117, 119, 121, 123–125, 127–134] and arthroscopic assessment (61%) [107, 108, 113, 116–122, 124–126, 130–133]. Most commonly used patient-reported outcomes are International Knee Documentation Committee (IKDC) score (36%), followed by a visual analogue scale (VAS) pain (39%) and Tegner activity scale (29%).

##### Adverse effects

None of the studies reported any severe adverse effects related to the MSC treatment. Two group reported minor adverse events including mild pain and effusion after the injections, which persisted for no more than 7 days [103, 114].

## Conclusions

There is a growing fascination with the role of mesenchymal stem cells in cartilage repair.

As early as the 1950s, Pridie showed fibrocartilaginous repair through subchondral drilling [135–137]. Initially, Pridie drilling was reported as a treatment for osteoarthritis [135, 138] and was often associated with many additional procedures such as synovectomy and trimming of osteophytes.

Since Pridie’s initial experiments, the process of marrow stimulation techniques or exposure of mesenchymal stem cells from cancellous bone has changed its guise on several occasions.

Ficat in 1979 described “Spongialization” in which the cancellous bed was exposed in 85 patients with chondral lesions of the patella with encouraging results [139]. Johnson et al. [140] described abrasion arthroplasty and encouraged its use especially in younger patients [141, 142]. Other authors had less positive outcomes [143–146]. Dandy wrote an entertaining article on abrasion arthroplasty where he highlighted that at least in the treatment of osteoarthritis, its effects could relate to the arthroscopic washout, rest or even the placebo effects of the charismatic surgeon [147]. The final evolution of marrow stimulation was the term “Microfracture” enabled by commercially manufactured bone picks used to breach the subchondral bone [8]. Marrow-stimulating technique procedures, in particular microfracture, are now considered the first-line treatment for full-thickness cartilage lesions and have demonstrated good to excellent results in 60–80% of patients [148, 149].

Cartilage repair has evolved from marrow stimulation techniques through to chondrocyte transplant and now stem cells at rapid pace. An ideal translational pipeline would demonstrate how in vitro data was used to inform a pre-clinical model, which would later form a phase I/IIa first-in-man study and subsequently a phase III clinical trial. This would of course be the safe and responsible method by which novel therapies are brought to the market.

This systematic review is the first of its kind to explore the full spectrum of evidence from in vitro studies, through animal studies to human clinical trials, and yet, we found little evidence of connectivity between in vitro, animal and then human work. In fact, we did not find a single group that had carried out and reported studies in all three categories.

Indeed, even from groups, which showed a seemingly hierarchical approach to translation, discrepancies became apparent. For example, Saw et al. from Korea used a pre-clinical goat model to repair cartilage defects using HA plus bone marrow-derived cells [150] and then moved into a first-in-man study, but in doing so, elected to change from bone marrow aspirate to peripheral blood and justified this change because it was easier to harvest peripheral blood than marrow [151].

There are several sources of cells that have been used in cartilage repair including bone marrow, peripheral blood, synovium, adipose tissue and umbilicus (Table 14) without any clear evidence of superiority of one over the other.

### One stage vs. two stages

As two stage procedures involving cell culture are expensive and cumbersome, there is an increasing push towards a single stage stem cell treatment. In this situation there is some supportive pre-clinical data [91, 95, 98, 152–154], but there does not appear to be a pre-clinical study that directly compares bone marrow concentrates against cultured MSCs.

Several groups have reported the use of bone marrow concentrates in clinical practice [116, 117, 127, 128, 130–132], in which the buffy coat is used containing the nucleated cells, of which a few will be stem cells.

Briefly, the patient has approximately 60 mL of bone marrow harvested from the iliac crest which is then spun down in a cell centrifuge (SmartPrep, Harvest Technologies Corp., USA, or IOR-G1, Novagenit, Mezzolombardo, TN, Italy) to provide 6 mL of concentrate containing nucleated cells. A small amount of the nucleated cells are then placed onto a hyaluronic acid membrane (Hyalofast, Fidia Advanced Biopolymers, Italy) or collagen membrane (IOR-G1, Novagenit, Mezzolombardo, TN, Italy) as a scaffold, which is then arthroscopically placed into the cartilage defect which had been pre-prepared using a burr or drill. The construct is then held with a platelet gel obtained from a harvest of 120 mL of patient’s venous blood taken the day before surgery (Vivostat system, (Vivolution, Denmark)) [118]. The results of the first 30 patients have been reported as showing improvements in MRI and arthroscopic appearance as well as clinical scores at 3 years follow-up [118].

This new technique is of course an evolution of the autologous matrix-enhanced chondrogenesis (AMIC) which used the stem cells from the adjacent marrow (and not pre-harvested bone marrow concentrates) within either collagen patches [155–157] or polyglycolic acid–hyaluronan-based scaffolds [158, 159].

There has also been a further step taken to avoid bone marrow harvest in which peripheral blood has been used in knee chondral lesions. In an RCT, arthroscopic subchondral drilling was followed by postoperative intra-articular injections of hyaluronic acid (HA) with and without peripheral blood stem cells (PBSC). Fifty patients were studied and randomised 1 week after surgery to receive either 8 injections of HA or 8 injections of HA plus PBSC. Those that underwent PBSC received stimulation with filgrastim, which contains recombinant human granulocyte colony-stimulating factor prior to harvest [106, 151]. At 18 month follow-up, they reported no adverse effects and improved MRI findings in the PBSC group compared to HA alone, took biopsies of 16 of the 25 patients in each group and claimed better tissue morphology in the PBSC group, as graded by the International Cartilage Repair Society Visual Assessment Scale II. Interestingly, however, the same group’s pre-clinical used bone marrow aspirates and not peripheral blood [150].

### Autologous vs. allogenic

There is an increasing interest in allogenic cells to avoid donor site morbidity and to reduce cost. The pre-clinical data with regards to allogenic cells is conflicting. One group showed promising results of allogenic MSCs in a rabbit model when compared to autologous cells, although numbers were small [160, 161]. Another group compared autologous chondroprogenitor cells and allogenic chondroprogenitor cells against controls in an equine model and reported inferior repair in the allogenic cell group [23]. Despite conflicting pre-clinical data, human studies using allogenic cells began in Korea in 2009. A phase I/IIa study to assess safety and efficacy of a combination of human umbilical cord blood-derived mesenchymal stem cells and sodium hyaluronate (CARTISTEM^®^ (MEDIPOST Co., Ltd., Korea)) was performed in knee chondral defects (NCT01041001). A parallel phase 3, open-label, multi-centre RCT comparing CARTISTEM^®^ and microfracture in knee chondral defects was carried out in Korea and the USA (NCT01733186). Results are still pending.

Another area of huge controversy is the actual dose of cells that should be used. In vitro between 50,000 cells/mL and 100 billion cells/ml have been studied. In pre-clinical animal studies, this ranged from 1000 to 1 billion cells/mL, and in human studies, the reported range has been 1.2 million cells/mL–24 million cells/mL.

It remains unclear what the most appropriate cell dose should be, with some groups reporting that a higher cell number leads to a better repair [52, 71, 87, 95, 162–164], but Zhao et al. [99] highlighted the limitation to cell saturation and survival, and thus, there may be a top limit to cell number that can be used to aid repair.

A multitude of methods for cell delivery have also been adopted, from direct joint injection or embedded in a plethora of scaffolds, such as type I collagen gels of porcine or bovine origin, ascorbic acid sheets or fibrin glues (Table 14).

In vitro and in pre-clinical studies, a plethora of growth factors have been studied including TGF-β1 and TGF-β2 and BMP-7 but none of these have been included in human clinical trials (Table 5).

It is clear that the relationship between cell passage, cell dose, the use of scaffolds and growth factors and the efficacy of MSC treatment is still to be established.

### Future

There is no question that the field of cartilage repair accelerates at rapid pace, and it is clear that the single stage procedures are likely to win over two stage procedures to save costs and reduce the burden on both provider and the patient. The reduction of donor site morbidity is a further driver helping direct progress.

The concept of cell banks of allogenic cells clearly meets all of the above criteria, but the lack of good supporting pre-clinical and long-term safety and efficacy data does little to pacify potential pitfalls of this direction. The fact that the phase 3 RCT of allogenic umbilical stem cells was allowed to be registered (NCT01041001) before the same group registered their phase I/IIa safety study (NCT01733186) intimates that sometimes clinical pace exceeds that of the regulators to lay down new ground.

Tools are likely to be introduced to the operating theatre that might improve the efficacy of treatment, such as fluorescence-activated cell sorting (FACS) machines which can isolate MSCs from the buffy coat of bone marrow aspirate by their cell surface markers. At present, this technology is expensive and complicated and ways to reduce cost and make the process simple are required before they could enter the operating theatre.

Induced pluripotent stem cells (iPSCs) are adult somatic cells that have been genetically reprogrammed to an embryonic stem cell-like state by being forced to express genes and factors important for maintaining the defining properties of embryonic stem cells [165].

These cells show unlimited self-renewal, and some in vitro studies have shown chondrogenic differentiation by iPSCs from human chondrocytes biopsied from osteoarthritic knees [166] and cartilage formation from human neural stem cells [167]. However, this work is at a very early stage, and aside from the ethical considerations, much research into control of cell phenotype and cell fate to alleviate concerns for cancer risk are required before this technology is ready to move into the pre-clinical and clinical realms.

In conclusion, this review is a comprehensive assessment of the evidence base to date behind the translation of basic science to the clinical practice of cartilage repair. We have revealed a lack of connectivity between the in vitro, pre-clinical and human data and a patchwork quilt of synergistic evidence. It appears that the drivers for progress in this space are largely driven by patient demand, surgeon inquisition, and a regulatory framework that is learning at the same pace as new developments take place. We strongly recommend funding body commission studies that have a clear translational purpose in order to drive the science towards patient benefit.

## Abbreviations

ACI: Autologous chondrocyte implantation
AMIC: Autologous matrix-enhanced chondrogenesis
AOFAS: American Orthopaedic Foot & Ankle Society
FACS: Fluorescence-activated cell sorting
HA: Hydroxyapatite
IKDC: International Knee Documentation Committee
iPSCs: Induced pluripotent stem cells
KOOS: Knee and Osteoarthritis Outcome Score
MACI: Matrix-induced autologous chondrocyte implantation
MeSH: Medical Subject Headings
MSC: Mesenchymal stem cells
OA: Osteoarthritis
PBS: Phosphate-buffered saline
PBSC: Peripheral blood stem cells
PRP: Platelet rich plasma
qPCR: Real-time polymerase chain reaction
RCT: Randomised controlled trial
VAS: Visual analogue scale
WOMAC: Western Ontario and McMaster Universities Arthritis Index
